# Perinatal Asphyxia Leads to PARP-1 Overactivity, p65 Translocation, IL-1β and TNF-α Overexpression, and Apoptotic-Like Cell Death in Mesencephalon of Neonatal Rats: Prevention by Systemic Neonatal Nicotinamide Administration

**DOI:** 10.1007/s12640-015-9517-0

**Published:** 2015-02-10

**Authors:** T. Neira-Peña, E. Rojas-Mancilla, V. Munoz-Vio, R. Perez, M. Gutierrez-Hernandez, D. Bustamante, P. Morales, M. A. Hermoso, P. Gebicke-Haerter, M. Herrera-Marschitz

**Affiliations:** 1 Millenium Institute BNI-Chile, Santiago, Chile; 2 Programme of Molecular & Clinical Pharmacology, ICBM, University of Chile, P.O. Box 70.000, Santiago 7, Chile; 3 Inmunology, ICBM, University of Chile, Santiago, Chile; 4 Universidad Bernardo OHiggins, Santiago, Chile; 5 Department of Psychopharmacology, Central Institute of Mental Health, J5, Mannheim, Germany; 6 c/o Goiny; Frihetsvägen 29, Järfälla, 17753 Järfälla, SE Sweden; 7 Av. Independencia, Independencia, Santiago, Chile

**Keywords:** Hypoxia–ischemia, Neonatal rat, Inflammatory mediators, Basal ganglia, Gene transcription

## Abstract

Perinatal asphyxia (PA) is a leading cause of neuronal damage in newborns, resulting in long-term neurological and cognitive deficits, in part due to impairment of mesostriatal and mesolimbic neurocircuitries. The insult can be as severe as to menace the integrity of the genome, triggering the overactivation of sentinel proteins, including poly (ADP-ribose) polymerase-1 (PARP-1). PARP-1 overactivation implies increased energy demands, worsening the metabolic failure and depleting further NAD^+^ availability. Using a global PA rat model, we report here evidence that hypoxia increases PARP-1 activity, triggering a signalling cascade leading to nuclear translocation of the NF-κB subunit p65, modulating the expression of IL-1β and TNF-α, pro-inflammatory molecules, increasing apoptotic-like cell death in mesencephalon of neonate rats, monitored with Western blots, qPCR, TUNEL and ELISA. PARP-1 activity increased immediately after PA, reaching a maximum 1–8 h after the insult, while activation of the NF-κB signalling pathway was observed 8 h after the insult, with a >twofold increase of p65 nuclear translocation. IL-1β and TNF-α mRNA levels were increased 24 h after the insult, together with a >twofold increase in apoptotic-like cell death. A single dose of the PARP-1 inhibitor nicotinamide (0.8 mmol/kg, i.p.), 1 h post delivery, prevented the effect of PA on PARP-1 activity, p65 translocation, pro-inflammatory cytokine expression and apoptotic-like cell death. The present study demonstrates that PA leads to PARP-1 overactivation, increasing the expression of pro-inflammatory cytokines and cell death in mesencephalon, effects prevented by systemic neonatal nicotinamide administration, supporting the idea that PARP-1 inhibition represents a therapeutic target against the effects of PA.

## Introduction

Obstetric complications are associated to neuropsychiatric disorders, with hypoxia as a recurrent co-factor, priming brain development by mechanisms not yet established (see Low 2004; Basovich 2010).

Delay in starting pulmonary ventilation at birth implies decrease of oxygen saturation in blood and its supply to the brain, which depends on aerobic metabolism for maintaining the respiratory chain and mitochondrial ATPase activity. Whenever hypoxia is sustained, there is a switch to glycolysis, a poor metabolic alternative, because of low stores of glucose in brain tissue and deficient ATP output by the glycolysis pathway, resulting also in lactate accumulation and acidosis. Prolonged hypoxia not only decreases gene expression and translation, but also increases the expression of sentinel proteins, such as poly(ADP-ribose) polymerases (PARPs) (Amé et al. 2004; Martin et al. 2005). Oxidative stress is inherent to re-oxygenation, resulting in over activation and inactivation of buffering enzymes (see Gitto et al. 2002). Indeed, in the clinical scenario, resuscitation implies hyperoxemia, free radical production and oxidative stress, worsening brain injury (Davis et al. 2004; Solberg et al. 2007; Kapadia et al. 2013).

Delayed cell death is an important endpoint of perinatal asphyxia (PA), associated to caspase-dependent and -independent mechanisms (Northington et al. 2001). Regionally selective nuclear fragmentation is observed in control and asphyxia-exposed rat pups, depending upon the stage of development and the analysed brain region, but apoptosis is sustained only in asphyxia-exposed animals (Dell’Anna et al. 1997).

The regional vulnerability of basal ganglia neurocircuitries to anoxia/ischemia has been pointed out by different groups (Pasternak et al. 1991; Pastuzko 1994; Cowan et al. 2003; Miller et al. 2005; Ferrari et al. 2011), reflecting either (i) the severity of the insult; (ii) the local metabolic imbalance during the re-oxygenation period and/or (iii) the developmental stage of the affected regions. Immaturity of a particular brain region plays a role, because the insult affects neuritogenesis and synaptogenesis (see Herrera-Marschitz et al. 2014).

PARP-1 is the most abundant and conserved member of a large protein superfamily, displaying a conserved catalytic domain for transferring ADP-riboses from NAD^+^ to glutamic and aspartic residues of the PARPs and their substrates, catalysing ADP-ribose polymerisation (pADPr). PARP-1 is involved in DNA repair, but overactivation of PARP-1 leads to NAD^+^ exhaustion and energy crisis (Berger 1985), inducing caspase-independent apoptosis (Jiang et al. 1996; Yu et al. 2002; see Hong et al. 2004). PARP-1 overactivation can promote nuclear factor κB (NF-κB) translocation (Hassa and Hottinger 1999) and expression of pro-inflammatory cytokines (Ullrich et al. 2001; Chiarugi and Moskowitz 2003; Hamby et al. 2007).

We report here that PA induces PARP-1 overactivation, in tandem with activation of pro-inflammatory signalling, increasing IL-1β and TNF-α mRNA levels and cell death in mesencephalon of rat neonates. Thus, PARP-1 overactivation is an early endpoint for PA, providing a target for preventing the effects elicited by metabolic insults at birth. As a proof of principle, we investigated the effect of nicotinamide (Virag and Szabo 2002) on PARP-1 activity, p65 nuclear translocation, inflammatory signalling and cell death, evaluated during the 24 h period following birth.

## Materials and Methods

### Animals

Wistar albino rats from the animal station of the *Molecular & Clinical Pharmacology Programme, ICBM*, Faculty of Medicine, University of Chile, Santiago, Chile, were used along the experiments. The animals were kept in a temperature- and humidity-controlled environment with a 12/12 h light/dark cycle and fed ad libitum, when not used for the experiments, monitoring permanently the well being of the animals by qualified personnel.

### Ethic Statement

All procedures were conducted in accordance with the animal care and use the protocol established by a Local Ethics Committee for experimentation with laboratory animals at the Medical Faculty, University of Chile (Comite de Bioetica Sobre Investigacion en Animals). The protocol was approved by the Ethics Committee (Protocol CBA#0447, FMUCH; August 4, 2011), which follows the Council Directive 2010/63EU of the European Parliament and the Council of 22 September 2010 on the protection of animals used for scientific purposes, as well as the Guidelines laid down by the NIH in USA. The protocol was further approved by the Comite Asesor de Bioetica, CONICYT the main granting institution in Chile (No. 018/FONDECYT/Medicina G1/615; 30 May 2012).

### Care and Final Cause of Each Animals

After using all pups, surrogate dams were kept alive for further programmed mating, delivery and surrogation (for 3–4 cycles), being then discharged according to established protocol, implying anaesthesia (cloral) and dislocation supervised by the Central Department of Experimental Animals of the Medical Faculty of the University of Chile. Experimental. Control animals were permanently monitored (on 24 h basis) regarding wellbeing, following the ARRIVE guidelines for reporting animal studies (www.nc3rs.org.uk/ARRIVE).

### Perinatal Asphyxia

Rats within the last day of gestation (G22) were anaesthetized, euthanized by neck dislocation and hysterectomised. One or two pups were removed immediately and used as non-asphyxiated caesarean-delivered controls (CS), and the uterine horns containing the remaining foetuses were immersed in a water bath at 37 °C for 21 min (AS). Following asphyxia, the uterine horns were incised and the pups were removed and stimulated to breathe. A group of CS (*n* = 5) and AS (*n* = 6) rats was euthanized immediately after delivery (0 h), in order to evaluate the effect of hypoxia, without any re-oxygenation. Otherwise, CS and AS were euthanized 1 h (CS *n* = 13; AS *n* = 13), 8 h (CS *n* = 24; AS *n* = 22) and 24 h (CS *n* = 26; AS *n* = 26) after delivered. After decapitation, the brain was rapidly removed and brain tissue was dissected on ice and stored at −80 °C, pending further experiments. Alternatively, the animals were first fixed with formalin. When required the pups were nursed by surrogate dams, and closely monitored for proper reception.

### Nicotinamide Treatment

One hour after birth, asphyctic and control rats were treated with a single dose of nicotinamide 0.8 mmol/kg (Sigma, St. Louis, MO, USA) (100 mg/kg, i.p.) or Saline (NaCl 0.9 %, i.p.) in a volume of 0.1 mL using a 0.5 mL insulin syringe (ANam *n* = 31; ASal *n* = 32; CNam *n* = 30; CSal *n* = 33). Thereafter, rats were euthanized and tissue was dissected in order to evaluate (i) PARP-1 activity; (ii) inflammatory-related molecules 2, 8 and 24 h post PA and (iii) cell death with a TUNEL assay 24 h post PA.

### Protein Extraction

For total protein extraction, pups were euthanized, 0–24 h post-birth. Mesencephalon and other suitable regions were dissected, frozen in liquid nitrogen and stored at −80 °C. Tissue was homogenized with a glass–glass homogenizer in ice-cold RIPA buffer (150 mM NaCl, 1 % Tritón X-100, 0.1 % SDS, 50 mM Tris pH 8; 0.5 % sodium deoxicolate) containing a Protease Inhibitor Cocktail Set III, EDTA-Free (Calbiochem, Darmstadt, Germany) and supplemented with 0.5 mM DTT, 0.1 mM Na_3_VO_4_, 100 µg/mL PMSF, 2 µg/mL leupeptin, 2 µg/mL aprotinin and 0.05 % Triton X-100. The homogenizate was incubated 30 min on ice, and centrifugated for 30 min at 13,500 rpm at 4 °C, recovering the supernatant.

### Nuclear and Cytoplasmatic Protein Extracts

Nuclear and cytoplasmatic proteins were extracted with a CelLytic™ NUCLEAR™ Extraction kit (Sigma, St. Louis, MO, USA) as described by the manufacturer. Briefly, tissue was homogenized in lysis buffer containing 0.1 M DTT and a protease inhibitor cocktail. The homogenizate was centrifugated at 11,000×*g* for 20 min at 4 °C. The supernatant containing cytoplasmatic proteins was recovered; the pellet resuspended in an extraction buffer, supplemented with 0.1 M DTT and a protease inhibitor cocktail. The homogenizate was agitated for 30 min and centrifugated at 21,000×*g* for 5 min at 4 °C, recovering the supernatant containing nuclear proteins.

### Protein Quantification

Protein levels were determined by the bicinchoninic acid (BCA) method (Pierce, BCA™ Protein Assay kit). BCA reacts with peptide bonds producing a purple end product. Equal amount (200 µL) of reagent A (1 % sodium BCA; 2 % sodium carbonate; 0.16 % sodium tartrate; 0.4 % NaOH and 0.95 % sodium bicarbonate) and reagent B (4 % cupric sulphate in 5× H_2_O) were mixed with 10 μL of each sample and incubated at 37 °C for 30 min. Samples were read at 562 nm in a Multi-Mode Microplate Reader (Biotek^®^ Instruments, Inc). A standard curve of absorbance versus micrograms of BSA protein was prepared, calculating the protein concentration for each sample.

### Western Blots Analysis

25 or 50 μL of protein extracts was mixed with sample buffer (125 mM Tris–HCl, pH 6.8; 2 % SDS, w/v; 20 % glycerol, w/v; 20 µg/µL bromophenol blue and 5 % β-mercaptoethanol), boiled for 5 min, separated by electrophoresis on a 8 % SDS-PAGE gel and blotted onto a BioTrace™ pure nitrocellulose membrane (Pall Corporation, Pensacola, FL, USA). Membranes were blocked with Tris-buffered saline (TBS) containing 0.1 % Tween-20 and 5 % (w/v) non-fat dry milk at room temperature for 1 h, and incubated with (i) mouse anti-pADPr (170–70 kDa) (3H2844) (sc-71848, Santa Cruz Biotechnology); (ii) goat anti-PARP-1 (113 kDa) (sc-F0908, Santa Cruz Biotechnology); (iii) Rabbit anti-p65 (65 kDa) (sc-372, Santa Cruz Biotechnology); (iv) Mouse anti-iKB-α (35–41 kDa) (sc-1643, Santa Cruz Biotechnology); (v) Mouse anti-β-actin (42 kDa) (Sigma A5316); (vi) Rabbit anti-histone H4 (13–15 kDa) (Abcam 1261) and (vii) mouse anti-α-tubulin (45 kDa) (Sigma T8203). Membranes were washed and incubated with HRP-conjugated secondary antibody in TBS at room temperature. The immune complexes were visualized with an enhanced chemiluminescent substrate, according to the instructions of the manufacturer (Perkin Elmer Life Sciences, Boston, MA), captured by a ChemiScope 3400 (Clinx Sciences Instruments Co, Ltd). Reactive bands were quantified by densitometric analysis with Photoshop software.

### Quantitative Real Time PCR (RT-qPCR)

Total RNA was extracted from brain samples using TRIzol (Invitrogen, USA). RNA was treated with TURBO DNA-free™ (Ambion Texas USA) and subsequently verified for optical density 260/280 absorption ratios. RNA integrity was evaluated by denaturing gel electrophoresis. CDNA was synthesized with an ImProm II reverse transcription system (Promega, Madison, USA), using oligo dT primers. RT-qPCR was performed using 2X Brilliant III SYBR^®^ Green QPCR Master Mix (Agilent Technologies, USA) in a MX3000 system (Stratagene, La Jolla, CA, USA). Primer sequences were designed with Primer3 software as follows:

**Table:** 

Primers	Forward sequence	Reverse sequence
p65	5′-ATAACTCGC CTGGTGACAGGAT-3′	5′-CTGAGAAGTCCATGTCCGCAAT-3′
IL-1β	5′-CTGCAGGCTTCGAGATGAACAA-3′	5′-TGTCCATTGAGGTGGAGAGCTT-3′
TNF-α	5′-GGCCAATGGCATGGATCTCAAA-3′	5′-AGC CTT GTC CCT TGA AGA GAA C-3′
GAPDH	5′-CCTGCC AAGTATGATGACATCAA-3′	5′-AGC CCA GGA TGC CCT TTA GT-3′

### Data were Analysed in Triplicates with MxPro Software, and Normalized to GAPDH mRNA Levels

#### Enzyme-Linked Assay (ELISA)

Quantification of IL-1β and TNF-α protein levels in brain tissue was performed by ELISA. The tissue was homogenized with a lysis buffer (25 mM Tris buffer, pH 7.4; 150 mM NaCl; 2 mM EDTA) containing a protease inhibitor cocktail (Calbiochem, Darmstadt, Germany). Samples were centrifugated at 15,000 g for 15 min at 4 °C, recovering the supernatant. Proteins were quantified as described above by the BCA method. A solid phase ELISA was performed using Quantikine^®^ ELISA, Rat IL-1β and Quantikine^®^ ELISA Rat TNF-α for measuring the respective cytokines, as described by the manufacturer. Briefly, 50 μL of standard, control or sample was added into microplates coated with a monoclonal antibody against IL-1β or TNF-α, incubated for 2 h at room temperature. Plates were washed and incubated with 100 μL of rat polyclonal anti-rat IL-1β or rat polyclonal anti-TNF-α conjugated with horseradish peroxidase for 2 h at room temperature. The plates were washed and incubated with 100 μL of the substrate solution containing hydrogen peroxide (H_2_O_2_) and tetramethylbenzidine as chromogen for 30 min at room temperature. The reaction was stopped and optical density was determined using a Multi-Mode Microplate Reader (Biotek^®^ Instruments, Inc) set at 450 nm.

The concentration of cytokines was calculated by extrapolation from a standard curve. Each sample was run by duplicated and the pg of cytokines was standardized by mg of protein for each sample.

#### Tissue Preparation for Evaluation of Apoptotic-Like Cell Death (TUNEL ASSAY)

Rat neonates (24 h after birth) were transcardially perfused with warm phosphate-buffered saline (PBS; 0.1 M; pH 7.4), followed by 4 % formalin solution in PBS. The brain was rapidly dissected and post-fixed for 24 h at 4 °C in the same fixative solution, and kept in 30 % sucrose for 48 h. Thereafter, the brains were serially cut into 20-μm-thick coronal cryostat sections. The sections were mounted on gelatinized slides and stored at −20 °C, pending further experiments.

Apoptotic-like DNA fragmentation was assessed with the TUNEL assay *Apop Tag* ^*®*^ Plus Peroxidase in Situ Apoptosis Detection kit (Millipore, Temecula, CA). Coronal sections were washed with PBS, and permeabilized with an ethanol:acetic acid (2:1) solution, quenched with 3 % H_2_O_2_ (v/v) for 5 min followed by 2 x washing with PBS. The terminal deoxynucleotidyl transferase (TdT) enzyme was added to the pre-equilibrated tissues and incubated for 1 h at 37 °C. Stop-buffer was added to the slide and agitated for 15 s and incubated for 10 min at room temperature. After washing three times with PBS for 1 min each, anti-digoxigenin peroxidase conjugate was added to the slides and incubated for 30 min. The slides were washed twice with PBS, and the freshly prepared peroxidase substrate 3,3′-diaminobenzidine was added to the slides for 7 min at room temperature, and then washed two times with distilled water. The sections were counterstained with 0.5 % Methyl Green (w/v) for 10 min followed by washing with water and then 100 % n-butanol. After 10 min, sections were dehydrated in xylene for 2 min and cover-slipped with Entellan (Merck, Darmstad, Germany). The stained sections were examined with a Nikon TS100 microscope. A preliminary observation under 10X was necessary for clearly identifying the selected brain areas. The number of TUNEL positive cells was quantified in 3–7 subareas (4.5 × 10^4^ μm^2^) from top to bottom for each section, from right to left hemisphere. The number of positive cells was expressed as the average of TUNEL positive cells per mm^3^.

#### Statistical Analysis

All data are expressed as mean ± SEM. The following are the experimental groups: (i) caesarean-delivered controls (CS); (ii) caesarean-delivered saline-treated controls (CSal); (iii) asphyxia-exposed (AS); (iv) asphyxia-exposed saline-treated (ASal); (v) caesarean-delivered nicotinamide controls (CNam) and (vi) asphyxia-exposed nicotinamide-treated (ANam) rat pups. Nicotinamide treatment was compared to the corresponding saline-treated controls, which is specifically indicated when required (see Materials and Method section). Pair-wise comparisons were evaluated with Student’s t-test, followed by Bonferroni correction to counteract the probability of false-positive results due to multiple comparisons, using a GraphPad Prism software. The significance level was set at *P* < 0.05.

## Results

### Perinatal Asphyxia

The experimental model implies a global non-invasive hypoxic insult occurring at the time when rats normally deliver (see Herrera-Marschitz et al. 2011). When possible, an Apgar scale is applied 1 h after delivery, to evaluate the severity of the insult. Compared to the controls (CS), the rate of survival was decreased following 21 min of PA (AS) (by ~40%). Surviving AS rats showed several abnormal parameters, indicating physiological and behavioural impairment, but were properly nursed by surrogate dams, independently upon nicotinamide or saline treatment.

### PARP-1 Levels: Estimation of Enzymatic Activity (Fig. 1)

The enzymatic activity of PARP-1 was estimated and expressed by the ratio of pADPr and PARP-1 (evaluated in the same membrane after stripping) levels, standardized by β-actin. PARP-1 activity was evaluated following hypoxia alone (immediately after delivery, 0 h) or after re-oxygenation was established (1–24 h), compared to the respective control rats.

**Fig. 1:**
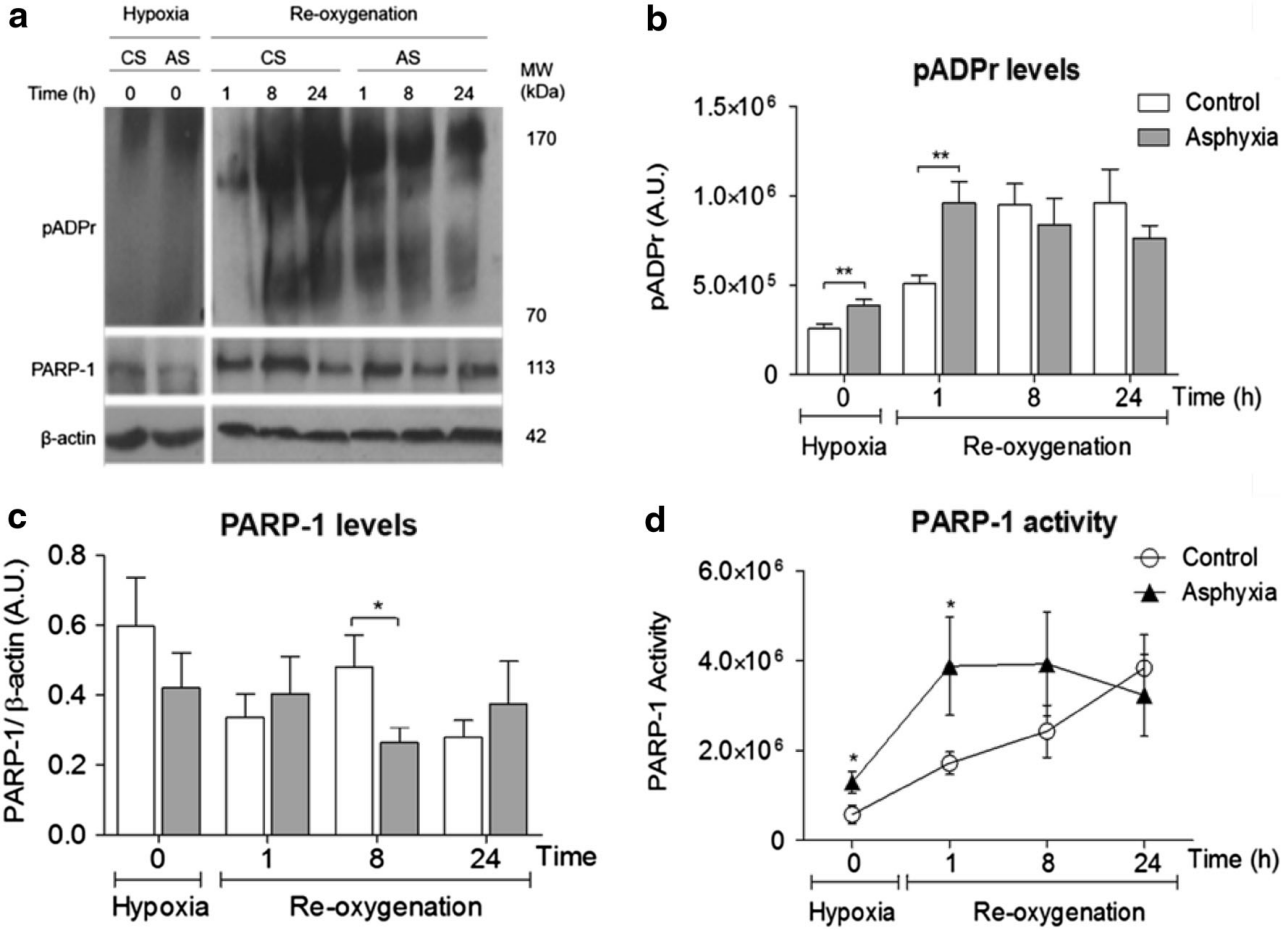
Effect of perinatal asphyxia on pADPr, PARP-1 levels and PARP-1 activity in mesencephalon of rat neonates, 0–24 h after delivery. Caesarean-delivered control (CS) and asphyxia-exposed (AS) rats were euthanized immediately after delivery (0 h), or following re-oxygenation (1–24 h), brain tissue sampled and treated for Western blots. **a** Representative immunoblots for pADPr (170–70 kDa), PARP-1 (113 kDa) and β-actin (42 kDa) levels following hypoxia and/or re-oxygenation. **b** pADPr levels (expressed as arbitrary units, A.U.) (CS *open columns*, AS *grey columns*). **c** PARP-1 levels, normalized to β-actin, A.U.). **d** PARP-1 activity (pADPr/PARP-1 ratio) (CS *open circles*, AS *filled triangles*). Pair-wise comparisons were analysed with Student *t* test (*p < 0.05, **p < 0.005; *n* = 5–7, for each condition and experiment)

Figure 1 shows pADPr, PARP-1 levels and PARP-1 activity measured in mesencephalon of CS and AS animals. In A, representative immunoblots of pADPr (170–70 KDa), PARP-1 (113 KDa) and β-actin (42 KDa) levels, in CS and AS animals, immediately (0 min) or 1–24 h after delivery, are shown. In B, pADPr levels are quantified as arbitrary units (A.U.), showing that pADPr levels increased after caesarean delivery, reaching a plateau 8 h after birth (white columns). In AS animals (grey columns), pADPr levels were increased, compared to CS animals, reaching a maximum 1 h after birth. In C, PARP-1 levels are standardized by β-actin levels, not showing any statistically significant differences among the groups, apart of a transient difference between AS versus CS, occurring at 8 h, when PARP-1 levels were decreased in AS animals. In D, PARP-1 activity is expressed as the ratio between pADPr and PARP-1, making evident that PARP-1 activity was increased in AS animals, 0–8 h after birth. No further differences were observed at 24 h.

### NF-κB Signalling Pathway Following PA and Re-oxygenation (Fig. 2)

IkBα and p65 levels were measured by Western blots in both CS and AS animals 1–24 h after delivery. IkBα was measured in total protein extracts, together with β-actin, while p65 was measured in cytoplasmic and nuclear extracts, together with α-tubulin and histone H4, for demonstrating the purity of the respective protein fractions. The novo synthesis of mRNA, including p65, IL-1β and TNF-α, was evaluated with RT-qPCR, 8–24 h after birth.

**Fig. 2:**
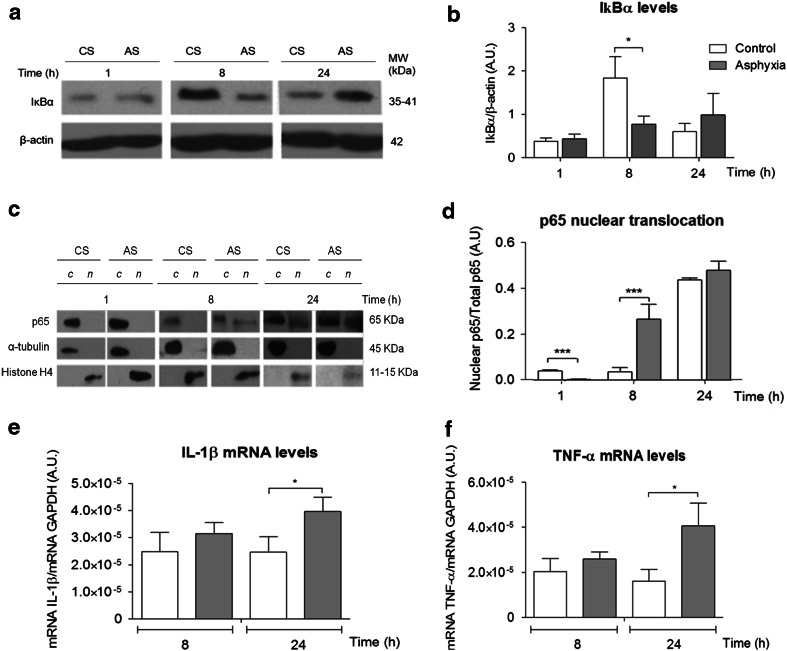
Effect of perinatal asphyxia on IκBα, nuclear translocation of p65, IL-1β and TNF-α mRNA levels in mesencephalon of rat neonates, 1–24 h after birth. Caesarean-delivered control (CS) and asphyxia-exposed (AS) rats were euthanized 1–24 h after delivery, and brain tissue sampled and treated for Western blots or RT-qPCR. **a** Representative immunoblots for IκBα (35–41 kDa) and β-actin (42 kDa) levels. **b** IκBα levels (normalized to β-actin; *A.U.* arbitrary units) (CS *open columns*, AS *grey columns*). **c** Representative immunoblots for p65 (65 kDa), α-tubulin (45 kDa) and histone H4 (15–11 kDa) levels, measured in cytoplasmic (*c*) and nuclear (*n*) protein extracts. **d** Nuclear p65 levels normalized to total p65. **e**, **f** IL-1β (E) and TNF-α **f** mRNA levels measured by RT-qPCR with specific primers; analysed in triplicates with MxPro software and normalized to GAPDH mRNA levels. Pair-wise comparisons analysed with Student *t* test (*p < 0.05, ***p < 0.0005; *n* = 5–7, for each condition and experiment)

Figure 2 shows IkBα and p65 levels measured by Western blots in mesencephalon of CS and AS animals. In A, representative immunoblots of IkBα (35–41 kDa), and β-actin (42 kDa) of CS and AS animals, 1–24 h after delivery, are shown. In B, IkBα normalized to β-actin (A.U.), showing that IκBα levels were increased in CS, but not in AS animals 8 h after delivery. IκBα levels were decreased in AS compared to that observed in CS animals. Otherwise, IκBα levels were rather stable along the experimental conditions, without any further statistically significant pair-wise differences along the evaluated periods. In C, representative immunoblots of p65 (65 kDa), α-tubulin (45 kDa) and histone H4 (15–11 kDa) in cytoplasmic (*c*) and nuclear (*n*) extracts are shown. In D, nuclear p65/total p65 in CS and AS animals, 1–24 h after delivery is shown. While decreased 1 h after delivery, nuclear p65 translocation increased > twofold 8 h after delivery in AS, compared to CS. In E and F, IL-1β and TNF-α mRNA levels were measured by RT-qPCR, normalized to GAPDH levels, 8 and 24 h after delivery, showing that both IL-1β and TNF-α mRNA levels were increased in AS compared to CS animals, 24 h after delivery.

### Apoptotic-Like Cell Death: Effect of Nicotinamide (Fig. 3)

The number of apoptotic nuclei was measured in sections from mesencephalon of asphyxia exposed and control animals with the TUNEL assay 24 h after delivery. Cells were counted on the stage of a Nikon microscope at 100 X on consecutive 20 μm sections, expressed as number of TUNEL-positive cells per mm^3^. A different series of asphyxia-exposed and caesarean-delivered control rats was treated either with a single dose of nicotinamide (0.8 mmol/kg, i.p.) (ANam, CNam) or with an equivalent amount of saline (0.1 mL, i.p.) (ASal, CSal), 1 h after delivery.

**Fig. 3:**
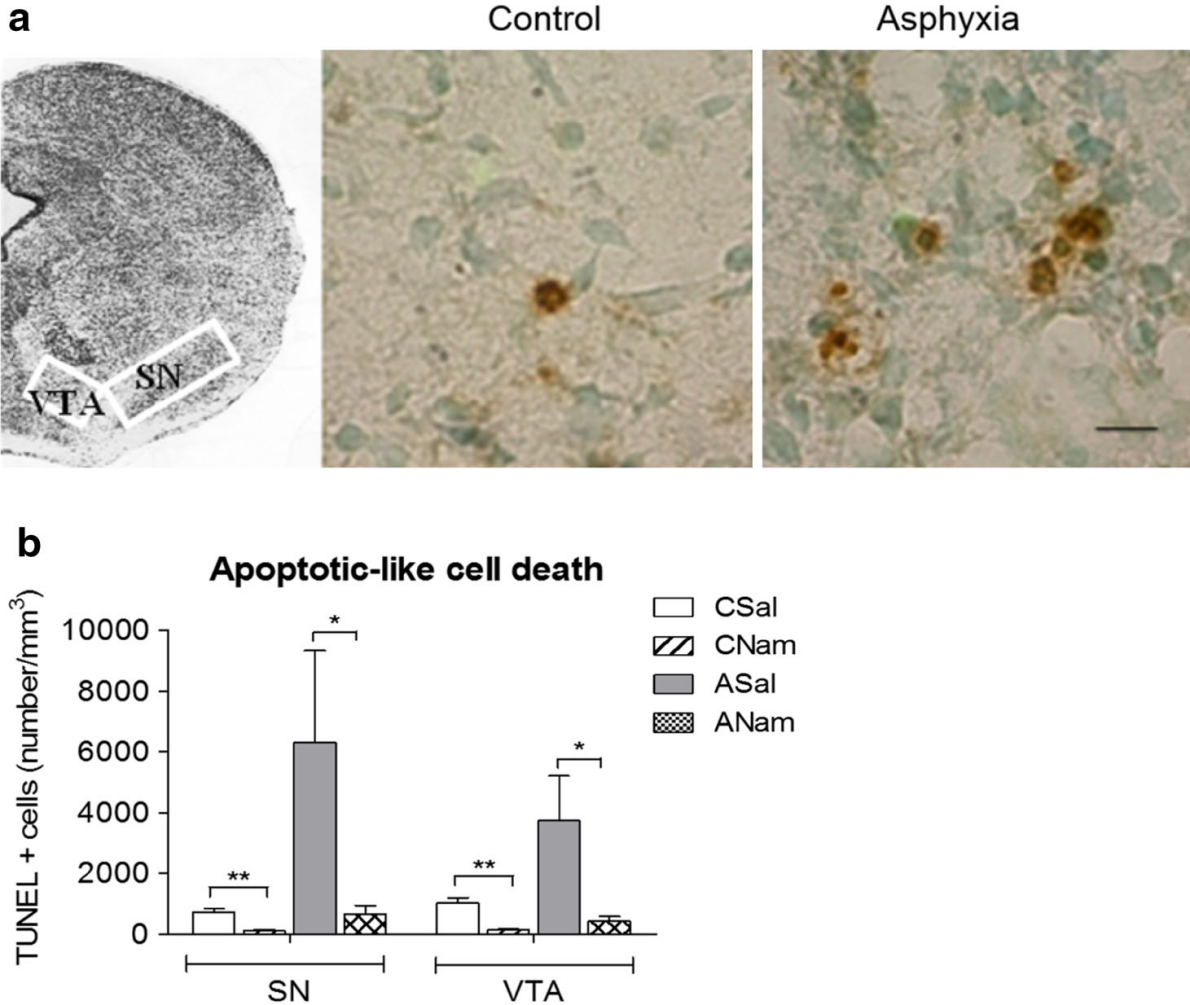
Effect of perinatal asphyxia on apoptotic-like cell death in mesencephalon of rat neonates, 24 h after delivery: prevention by systemic nicotinamide treatment. Caesarean-delivered control (CS) and asphyxia-exposed (AS) rats were anaesthetized and transcardially perfused with buffered saline and a formalin solution 24 h after delivery. DNA fragmentation was evaluated in coronal brain sections (20-μm thick) with the TUNEL assay, counterstained with methyl green. The number of TUNEL-positive cells per mm^3^ was determined in substantia nigra (SN), and ventral tegmental area (VTA). **a** Representative microphotographs, from SN of control and asphyxia-exposed animals; TUNEL-positive cells are *brown* (*arrows*) (bar 10 μm). Inset shows a section from Foster atlas (1998), indicating the sampling area (*white rectangles*). **b** TUNEL-positive cells were manually counted on the stage of a Nikon TS100 microscope (magnification ×100), expressed as number of TUNEL-positive cells/mm^3^ in CS and AS animals, or in parallel animal series following a single dose of nicotinamide (0.8 mmol/kg, i.p.) (CNam, ANam) or saline (0.1 ml, i.p.) (CSal, ASal) 1 h after delivery; also fixed at 24 h (CSal *open columns*; CNam *dashed columns*, ASal *grey columns*, ANam *doubled dashed columns*). Pair-wise comparisons analysed with Student *t* test (*p < 0.05; *n* = 4–5, for each condition)

Figure 3 shows the quantification of apoptotic-like cell death, evaluated with the TUNEL assay. In A, representative microphotographs illustrating TUNEL-positive cells in mesencephalon of CS and AS animals, 24 h after birth. In B, the number of TUNEL-positive cells was significantly increased in AS, compared to CS rats, both in substantia nigra (SN) and ventral tegmental area (VTA). Nicotinamide treatment decreased apoptotic-like cells in mesencephalon of asphyxia-exposed animals (ANam versus ASal), both in SN and VTA. A decrease of apoptotic-like cells was also observed in control animals treated with nicotinamide (CNam versus CSal).

### Effect of Nicotinamide Treatment on PARP-1 Activity (Fig. 4)

The effect of nicotinamide on PARP-1 activity was evaluated at an earlier stage (2 h after birth) than that for inflammatory proteins (8–24 h) or apoptosis (24 h), to pick up a sequential effect leading to the endpoint.

**Fig. 4:**
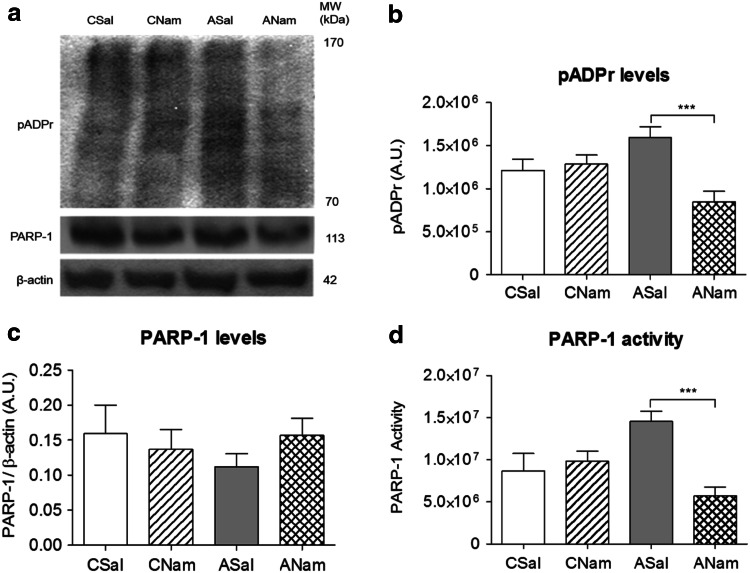
Effect of nicotinamide or saline on PARP-1 activity in mesencephalon of asphyxia-exposed and control rats. Caesarean-delivered control (CS) and asphyxia-exposed (AS) rats were treated 1 h after birth with a single dose of Nam (0.8 mmol/kg, i.p.) (CNam, ANam) or saline (0.1 ml, i.p.) (CSal, ASal), and euthanized 2 h after delivery. Brain tissue sampled and treated for Western blots. pADPr and PARP-1 protein levels were measured in total protein extracts. PARP-1 activity was estimated as the pADPr/PARP-1 ratio. **a** Representative immunoblots for pADPr, PARP-1 and β-actin levels; **b** pADPr levels (expressed as arbitrary units, A.U.); **c** PARP-1 levels (normalized to β-actin, A.U.); **d** PARP-1 activity (pADPr/PARP-1 ratio) (CSal *open columns*, CNam *dashed columns*, ASal *grey columns*, ANam *double dashed columns*). Pair-wise comparisons analysed with Student *t* test (*p < 0.05, **p < 0.005, ***p < 0.0005; *n* = 4–5, for each condition and experiment)

Figure 4 shows the effect of nicotinamide (CNam, ANam), or saline (CSal, ASal) on pADPr (A, B), PARP-1 levels (C) and PARP-1 activity (D) 2 h after birth. Nicotinamide decreased pADPr levels (B) and PARP-1 activity (D) (ANam versus ASal). No differences were observed in PARP-1 levels (C) between any of the groups.

### Effect of Nicotinamide on the Inflammatory Cascade Elicited by PA (Fig. 5)

**Fig. 5:**
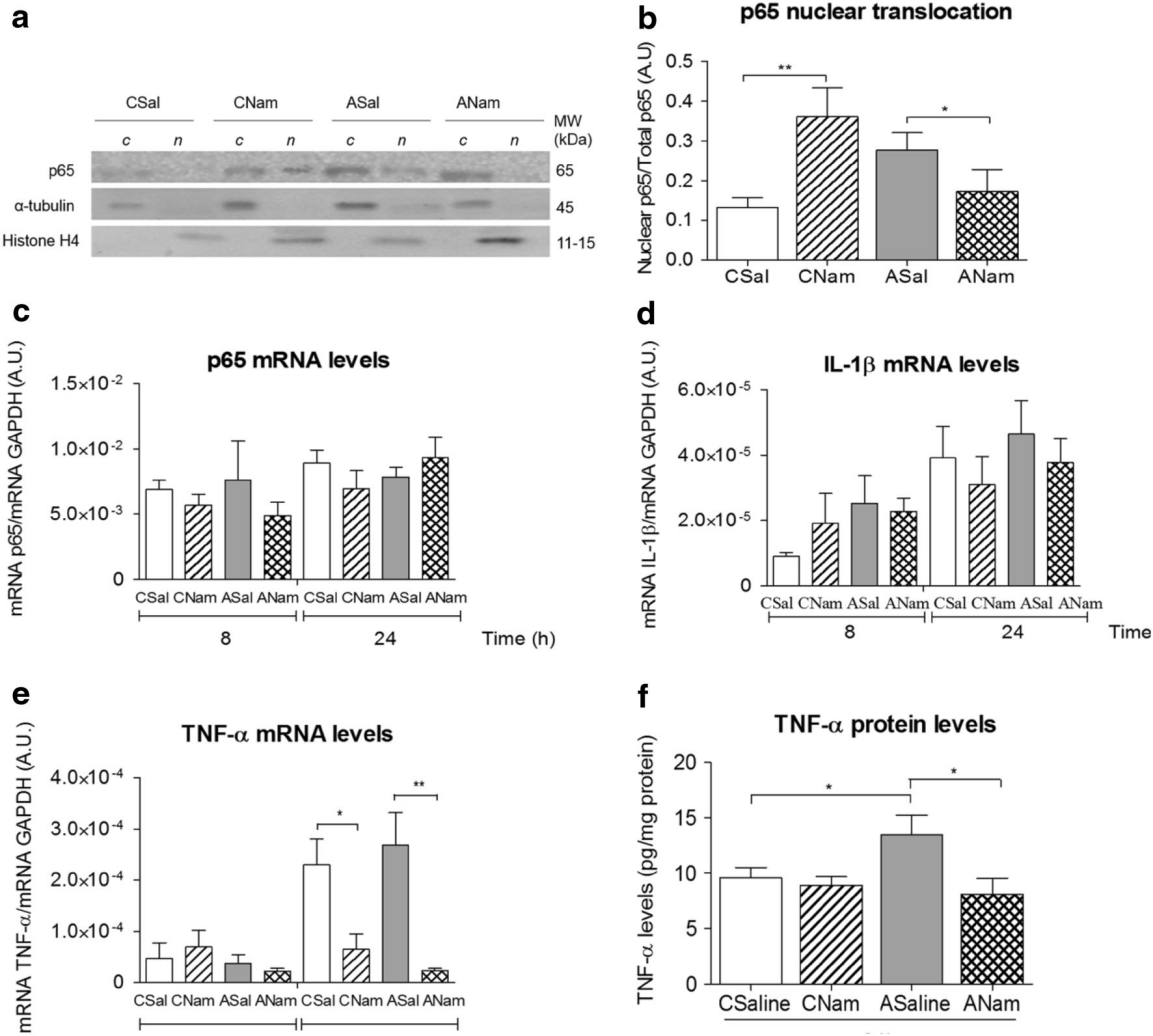
Effect of nicotinamide or saline on p65 translocation; on p65, IL-1β and TNF-α mRNA levels, and on ELISA TNF-α protein levels in mesencephalon of asphyxia-exposed and control rats. Caesarean-delivered control (CS) and asphyxia-exposed (AS) rats were treated with a single dose of nicotinamide (0.8 mmol/kg, i.p.) (CNam, ANam) or saline (0.1 ml, i.p.) (CSal, ASal), 1 h after delivery, euthanized at 8 or 24 h after birth. Selected brain tissue was treated for Western blots, RT-qPCR or ELISA. **a** Representative immunoblots for p65, α-tubulin and histone H4 levels, measured in cytoplasmic (*c*) and nuclear (*n*) protein extracts. **b** Nuclear p65 levels normalized to total p65. **c** De novo synthesis of p65, **d** IL-1β and **e** TNF-α, measured by RT-qPCR 8 h and/or 24 h after delivery (data analysed in triplicates with MxPro software, normalized to GAPDH mRNA levels). **f** TNF-α protein levels measured in total protein extracts with Quantikine^®^ ELISA Rat (normalized by mg of protein for each sample, in duplicate) (CSal *open columns*, CNam *dashed columns*, ASal *grey columns*, ANam *doubled dashed columns*). Pair-wise comparisons analysed with Student *t* test (*p < 0.05, **p < 0.005)

Figure 5 shows the effect of nicotinamide on p65 translocation and on p65 mRNA levels in mesencephalon from control and asphyxia-exposed animals, 8 and 24 h after birth. In A, representative immunoblots of p65 (65 kDa), α-tubulin (45 KDa) and histone H4 (15–11 kDa) in cytoplasmic (*c*) and nuclear (*n*) extracts are shown. In B, quantification of nuclear p65/total p65 in CSal, CNam, ASal and ANam animals is shown. Nicotinamide increased p65 translocation in control (CNam versus CSal), but decreased p65 translocation in asphyxia-induced (ANam versus ASaline) animals, 8 h after delivery, but no statistically significant level was reached. In C, p65 mRNA/GAPDH mRNA levels in saline and nicotinamide-treated asphyxia-exposed and control animals 8–24 h after delivery, and in D IL-1β mRNA, were evaluated under the same conditions. No differences were observed among any of the conditions. As shown in E, TNF-α mRNA levels were, however, significantly decreased by nicotinamide, both in control and asphyxia-exposed animals, 24 h after delivery. When TNF-α protein levels were measured with ELISA, it was evident that asphyxia increased TNF-α levels 24 h after delivery, which was reversed by nicotinamide (Fig. 5F). No effect was observed on IL-1β protein levels (data not shown).

## Discussion

PA implies interruption of oxygen availability, leading to death if oxygenation is not promptly re-established. In the present study, ready to birth rat foetuses (removed by caesarean at G22) were immersed into a water bath for 21 min, resulting, when delivered and stimulated to breathe, in a significant decrease of the survival rate. As characterized by an Apgar scale, PA surviving pups showed several signs of physiological impairment, mainly affecting cardiovascular and CNS-dependent functions. When assayed 24 h after birth, it was clear that the insult led to TUNEL-positive cell death in different brain regions, mainly in mesencephalon.

Cell death can be a direct result of the shifting from aerobic to inefficient anaerobic metabolism produced by asphyxia, generating long-lasting metabolic by-products and acidosis. Otherwise, cell death can be the consequence of re-oxygenation, which is a requirement for survival, leading to hyperoxemia, increasing ROS levels (Mishra et al. 2003; Strosznajder et al. 2010) and affecting CNS development, since brain tissue is particularly vulnerable to ROS, because of its high metabolic rate and low antioxidant defences at birth (see von Bernhardi et al. 2010). Furthermore, oxidative stress implies DNA damage, and activation of sentinel proteins for protecting the genome.

PARP-1 is in the front of sentinel proteins involved in the maintenance of chromatin integrity, recruiting the DNA repair machine through poly-ADP ribosylation of histones, relaxing the chromatin and modulating factors with high affinity for pADPr, therefore demanding and exhausting NAD^+^ sources, worsening the energy crisis (Berger 1985), leading to caspase-independent apoptosis, via translocation of mitochondrial pro-apoptotic proteins (Jiang et al. 1996; Yu et al. 2002). Thus, it has been proposed that PARP-1 is involved in the long-term effects produced by PA (Martin et al. 2005; Klawitter et al. 2006; see Herrera-Marschitz et al. 2011), making relevant to monitor PARP-1 levels and PARP-1 activity at early stages, when the babies are recovering from PA.

PARP-1 levels were measured in mesencephalon, immediately after asphyxia, or 1–24 h after delivery, obtaining rather variable results without a revealing pattern associated to PA. However, when PARP-1 activity was estimated by the ratio of pADPr/PARP-1 levels, it was evident that the insult produced a significant increase of PARP-1 activity in mesencephalon 1–8 h after PA.

Several studies have pointed out the role of PARP-1 during inflammatory processes, because PARP-1 positively regulates NFκB activation via (i) IKKγ, the kinase that phosphorylates IκBα, inducing its degradation; (ii) production of transcriptionally active subunits of the NFκB complexes, mainly the dimer p50/RelA (p65) and/or (iii) via pADP ribosylation of p65, which is translocated to and retained into the nucleus (Chiarugi and Moskowitz 2003; see Rosado et al. 2013). Thus, it was found here that IκBα levels were decreased in the cytosol compartment of mesencephalic cells 8 h after PA, compared to that observed in caesarean-delivered control siblings. Furthermore, p65 was translocated to the nucleus, increasing 1 h, but decreased 8 h after PA.

We further investigated whether p65 translocation led to *de novo* synthesis of the pro-inflammatory signalling, IL-1β and TNFα, monitored along the same experimental conditions with RT-qPCR and ELISA. It was found that IL-1β and TNFα mRNA protein levels were increased 24 h after the insult.

PARP-1 overactivation has been considered to be an endpoint for several metabolic insults including PA, leading to the hypothesis that PARP-1 inhibition constitutes a pharmacological target for preventing the long-term effects elicited by asphyctic/ischemic insults (see Kauppinen and Swanson 2007). Thus, we tested here the effect of the PARP-1 inhibitor nicotinamide, the amide form of nicotinic acid (vitamin B3/niacin), on cell death evaluated 24 h after PA and on PARP-1 activity and the inflammatory cascade initiated by p65 nuclear translocation at the 2–24 h period after birth. Nicotinamide interacts with the binding site for the nicotinamide moiety of NAD^+^ at the active site of PARP, inducing end product enzymatic inhibition (see Virag and Szabo 2002). Nicotinamide or saline was administered 1 h post delivery to PA and control pups. It was found that nicotinamide significantly decreased cell death induced by PA, evaluated 24 h after birth.

Delay in starting pulmonary ventilation at birth implies decrease of oxygen saturation in blood and oxygen supply to the brain. In response to energy deficit, blood flow is redistributed to the heart, brain and adrenal glands to ensure oxygen supply to these vital organs. This redistribution occurs at the expense of reduced perfusión of kidneys, gastrointestinal tract, muscles, skeleton and skin (Peeters et al. 1979; Lubec et al. 1997; see Jensen and Berger 1991; Berger and Garnier 2000). In the brain, there is also a redistribution of blood flow, favouring the brain stem at the expense of the cortex (Lou et al. 1985), and probably, further re-compartmentalisation occurs depending upon the developmental stage of particular regions (see Herrera-Marschitz et al. 2011, 2014). During re-oxygenation, extracellular levels of glutamate are increased, enhancing the activation of Na^+^/K^+^ ATPase, increasing further ATP consumption. Extracellular glutamate levels overpass the buffer capacity of astrocytes, resulting in overactivation of glutamate receptors, mainly of the *N*-methyl-d-aspartate (NMDA) subtype, increasing Ca^2+^ conductance and further improper homeostasis, therefore, glutamate antagonism has been proposed as a therapeutic intervention, with rather mild effects (see Engidawork et al. 2001). The metabolic crisis is probably sustained by over expression of alternative metabolic pathways, prolonging the energy deficit and oxidative stress, leading to cell death, which can occur via several mechanisms. The prevalent type of delayed cell death in the perinatal brain is apoptosis, which is mediated by caspase-dependent and caspase-independent mechanisms (Northington et al. 2001; see Morales et al. 2008). Indeed, not only pro-apoptotic proteins have been observed to be increased following PA, including Bcl-2 associated X (BAX), and Bcl-2 associated death (BAD) factors, but also anti-apoptotic proteins, including Bcl-2, ERK2 and bFGF, suggesting the activation of neuroprotective and repair pathways (Morales et al. 2005, 2008). Extensive and regionally selective nuclear fragmentation has been observed in control and asphyctic rat pups, depending upon the stage of development and the analysed brain region (Dell’Anna et al. 1997). In the present report, we focused on a short period, 24 h, in order to pick up metabolic events leading to an early relevant endpoint, finding increased apoptotic-like cell death in mesencephalon, both in SN and VTA. Also hippocampus and telencephalon have been shown to be relevant targets, but only when assayed 1 week or longer periods after birth (Dell’Anna et al. 1997; Morales et al. 2010).

Several assays have been developed to measure PARP-1 enzymatic activity (see Allende-Castro et al. 2012). We chose here an *in vivo* assay based on pADPr accumulation, estimating the ratio between pADPr and PARP-1 levels (ratio end product/enzyme level), revealing an early, regionally specific profile, indicating that PARP-1 activity is indeed increased by PA, further demonstrated by the effect of nicotinamide, reversing the overactivation of PARP-1 2 h after birth.

Several studies have suggested further that PARP-1 inhibition protects against acute and chronic inflammation, since PARP-1 positively regulates NFκB activation, via IKKγ, the kinase phosphorylating IκBα, inducing its degradation, also favouring pADPribosylation of the p50/p65 dimer and its ulterior translocation (see Rosado et al. 2013; also Zerfaoui et al. 2010). We measured here IκBα, and p65 levels in cytoplasma and nuclear protein fractions, and transcription of pro-inflammatory signals, finding that IL-1β and TNF-α mRNA levels were increased in mesencephalon of asphyxia-exposed animals, together with a decrease of IκBα levels and p65 nuclear translocation, effects that were prevented by nicotinamide, mainly on TNF-α, at mRNA and protein levels, monitored with RT-qPCR and ELISA, respectively. The NFκB cascade leads to IL-1β and TNF-α transcription and other inflammatory genes, such as IL-6. IL-1β is synthesized as a pro-IL-1β, which has to be cleavaged to generate mature IL-1β. In the present study, no effect was observed on IL-1β protein levels, perhaps because IL-1β maturation implies activation of caspase-1 and a multiprotein cytoplasmatic complex named inflammasomes, associated to neurotoxicity (see Schroder and Tschopp 2010). TNF-α was monitored at both mRNA and protein levels, revealing a significant increase in mesencephalon of asphyxia-exposed animals 24 h after the insult, prevented by nicotinamide. A similar effect has been observed in hippocampus, also prevented by nicotinamide, but mainly 8 h after the insult (Neira-Pena et al. in preparation), suggesting the involvement of different protein cascades (see Palladino et al. 2003).

Interestingly, exposure of embryonic rat mesencephalic neuronal cultures to TNF-α resulted in a dose-dependent decrease of the number of tyrosine hydroxylase (TH) immunoreactive cells (see Kraft and Harry 2011). In humans, a relationship between IL-1β, IL-6 and TNF-α serum level and the outcome of PA suffering infants has been established. Infants deceased before the first year of life, or diagnosed as suffering of cerebral palsy, presented elevated levels of pro-inflammatory cytokines, compared to that shown by infants with a normal outcome (Foster-Barber et al. 2001), suggesting that pro-inflammatory cytokine levels provide a predictive value for the final neurological outcome (Aly et al. 2006).

## Conclusions

PA leads to death whenever re-oxygenation is not promptly re-established. In rats, it leads to a significant decrease of the survival rate. We show here that surviving pups show an increase of TUNEL-positive cell death evaluated 24 h after birth. Cell death implies DNA damage, and activation of sentinel genome protecting proteins. PARP-1 is in the front of sentinel proteins involved in the maintenance of chromatin integrity, demanding and exhausting available NAD^+^ pools, worsening the energy crisis. PARP-1 activity was monitored at early stages after birth, in a brain region showing increased cell death. When estimating PARP-1 activity by the ratio of pADPr/PARP-1 levels, it was evident that the insult led a significant increase of PARP-1 activity in mesencephalon. The increase of PARP-1 activity was associated to p65 translocation and *de novo* synthesis of pro-inflammatory signalling, IL-1β and TNFα. Nicotinamide prevented PA-induced PARP-1 overactivity, p65 translocation, and reversed the increase of inflammatory signalling, mainly TNF-α. Thus, the present results show that PA leads to overactivation of PARP-1, increasing the expression of pro-inflammatory cytokines and cell death, effects prevented by systemic neonatal nicotinamide administration.
